# A Movement-Assisted Deployment of Collaborating Autonomous Sensors for Indoor and Outdoor Environment Monitoring

**DOI:** 10.3390/s16091497

**Published:** 2016-09-14

**Authors:** Ewa Niewiadomska-Szynkiewicz, Andrzej Sikora, Michał Marks

**Affiliations:** 1 Research and Academic Computer Network, Warsaw 01-045, Poland; ewan@nask.pl (E.N.-S.); asikora@nask.pl (A.S.); 2 Institute of Control and Computation Engineering, Warsaw University of Technology, Warsaw 00-665, Poland; ens@ia.pw.edu.pl

**Keywords:** ad hoc network, MANET, self-organizing network, mobility model, deployment model

## Abstract

Using mobile robots or unmanned vehicles to assist optimal wireless sensors deployment in a working space can significantly enhance the capability to investigate unknown environments. This paper addresses the issues of the application of numerical optimization and computer simulation techniques to on-line calculation of a wireless sensor network topology for monitoring and tracking purposes. We focus on the design of a self-organizing and collaborative mobile network that enables a continuous data transmission to the data sink (base station) and automatically adapts its behavior to changes in the environment to achieve a common goal. The pre-defined and self-configuring approaches to the mobile-based deployment of sensors are compared and discussed. A family of novel algorithms for the optimal placement of mobile wireless devices for permanent monitoring of indoor and outdoor dynamic environments is described. They employ a network connectivity-maintaining mobility model utilizing the concept of the virtual potential function for calculating the motion trajectories of platforms carrying sensors. Their quality and utility have been justified through simulation experiments and are discussed in the final part of the paper.

## 1. Introduction

Progress in hardware and networking technologies enables large-scale deployment of collaborating sensing devices and the creation of modern data acquisition systems that can greatly enhance the capability to sense and control physical environments. The potential applications contain a comprehensive surrounding monitoring, unmanned space exploration, objects tracking, surveillance systems, etc. Indeed, recently, a tremendous interest can be seen in the design and development of wireless sensor networks (WSNs), i.e., dynamically-configurable, self-organizing computer networks composed of numerous smart, embedded sensing devices that communicate wirelessly sharing the same radio channel. Rising demand for the capabilities of sensing systems, the lack of fixed network infrastructure and the limited energy and computation resources of their components provoke a broad spectrum of hardware and software engineering challenges involving high quality and secure communication, localization, optimal deployment, energy-efficiency, self-operability, scalability and performance. To meet these needs, a variety of methods is used and implemented, which results in the development of novel communication protocols, data acquisition algorithms, localization schemes and deployment techniques.

WSNs utilize an ad hoc networking, a new paradigm of communication where all wireless devices (network nodes) communicate with each other in a collaborative way to achieve a common goal, usually without central management. Nodes (data sources) transmit data to devices located within their transmission range. For communication with a base station (data sink) and nodes beyond the transmission range, the relay nodes are used. The lack of fixed network infrastructure components allows creating unique topologies and enables the dynamic adjustment of sensing devices’ operation to the current requirements. However, to fulfill sensing tasks, a full coverage of a region of interest (ROI) and permanent connection between sensing devices and a base station are usually required. A weak deployment of sensors may result in sensing holes and losses of connectivity or the redundancy of coverage, which causes redundant data in the network and usually wastes energy resources. On the other side, the obligation of providing high coverage of ROI and network connectivity leads to the necessity in dealing with the network construction costs’ trade-off, as aggressive consolidation may lead to performance degradation. Consequently, an optimal deployment of sensors has received significant attention in the recent years. A variety of approaches and techniques has been proposed and presented in the literature [1,2].

In order to meet the challenging goals of novel indoor and outdoor sensing systems, the mobility of sensors is often leveraged for the deployment [3,4,5,6]. Sensors can be mounted on mobile platforms, i.e., unmanned vehicles, mobile robots or drones, and moved to desirable positions. It is obvious that mobility implies an additional complexity layer. However, the complexity of WSN design, implementation and management is compensated by a number of benefits. Endowing network nodes with mobility drastically expands WSN capabilities. Mobility allows one to detect the lack of deployment objectives, improve coverage and communication connectivity, even decreasing the number of employed sensing devices. A large number of measurement targets can be handled with a smaller number of migrating sensors.

Although numerous movement-assisted deployment strategies have been investigated and are described in the literature, the development of a scalable and robust technique for sensors’ placement to achieve the optimal sensing coverage and ensure satisfactory communication connectivity is still a challenging task, especially in the case of an unknown and changing region of interest. The aim of this paper is to present the application of numerical optimization, specialized heuristics and computer simulation to movement-assisted deployment of sensors in a sensing area. We consider networks with self-configuring capabilities that comprise multiple autonomous mobile devices equipped with various detectors and radio transceivers. We have developed a family of algorithms for forming adaptive and coherent wireless sensor networks for monitoring purposes. Both pre-configuring and self-configuring topologies have been considered. In general, the proposed algorithms share similar goals with many other solutions described in the literature. We focus on the development of an optimal sensing network formed by mobile sensors for outdoor and indoor environment monitoring, gas cloud detecting and surroundings, human existence detecting in the case of a disaster, rescue action supporting, etc. The aim is to determine the minimum number of sensors needed to be placed to achieve an optimal sensing coverage, maintain communication with the base station and minimize the energy usage and time for carrying sensors. Furthermore, we assume that our sensing networks should dynamically adapt to changing environments and fully cover a region of interest while new events occur, without internal sensing holes. The novelty in our approach is the simulation-based computing scheme for the calculation of mobile sensors’ displacement ensuring permanent connectivity with the base station. It employs numerical optimization to calculate optimal positions of sensors and the concepts of an artificial potential field and a particle-based mobility to calculate collision-free traveling patterns for mobile platforms carrying sensors. Multiple simulations were performed to show the effectiveness of the proposed deployment strategies considering WSN sensing coverage and connectivity. We assessed the quality of the presented deployment strategies based on the results obtained for outdoor and indoor regions of interest.

The remainder of the article is organized as follows. The related work is reviewed in Section 2. A formal statement of a problem of sensing network design and the performance measures are provided in Section 3. The proposed deployment strategies and the model for motion trajectories’ calculation are described in Section 4. The results of simulation studies are presented and discussed in Section 5. Finally, we conclude the paper in Section 6.

## 2. Deployment Strategies in a WSN

Effective deployment of sensing devices is a fundamental issue in implementing a multi-hop wireless sensor network that enables the monitoring of a bounded region of interest and transmitting measurements to the base station. The deployment model determines the types, numbers and positions of devices in order to create a powerful system fulfilling the requirements of a given application scenario. Various models have been developed and described in the literature. Their performance can be evaluated using multiple metrics, such as coverage, connectivity, transmission quality, cost, etc. A brief overview of the deployment strategies is presented in the following subsections. The attention is focused on models for WSNs composed of mobile sensors.

### 2.1. Classification of Deployment Strategies

The deployment strategies can generally be divided into:

- random dropping,
- stationary deployment,
- movement-assisted deployment.

Random dropping of sensing devices is a widely-used deployment strategy, especially in disaster areas, wilderness, harsh fields or toxic urban areas, where sensors cannot be deployed manually. In general, due to the uneven distribution, the unknown and uncontrolled location of each device and the significant risk of unpredictable node failure, it cannot be expected that sensors are placed in a desired way. Moreover, sensor devices can fail at runtime for various reasons, such as damaging events, hardware effects, power depletion and degrading coverage. Consequently, the final network cannot satisfy the requirements for connectivity, cooperation and coverage, no matter how many devices are dropped. Dropping sensors in groups and a multi-stage strategy, where sensors are deployed iteratively taking into account the quality of previous deployments, are proposed for coverage improvement [7,8,9]. However, in general, random dropping can be used at initiation, but is not always a sufficient solution.

In the case of a known, bounded sensing space, it is possible to calculate the optimal locations of sensing devices to obtain a good coverage and connectivity network [10]. All spatial positions of sensors are a priori determined. The key problems are how many sensors are sufficient for covering the region of interest, how to ensure a coverage of good compactness and the satisfied communication connectivity in the case of a sufficient number of sensors. Various solutions are presented and discussed in the literature. The most popular approach is to create regular grid topologies. The common idea of other methods is formulating a linear or a nonlinear optimal covering problem solved by optimization solvers.

Recently, in many applications, movement-assisted sensor placement is investigated. The lack of the fixed spatial positions of sensors allows creating unique topologies (optimal at a given time) and enables the network to be dynamic and flexible. Mobile sensors can be used to cover the region of interest and/or to improve the coverage by detecting and covering holes in a network, replacing failed nodes and restoring connectivity. However, for the movement-assisted deployment, several basic issues must be solved. The most important ones are: localization in the case of a dynamically-changing network topology, maintaining communication connectivity and energy conservation. The GPS systems or specialized location schemes [11,12,13] are required to track the spatial positions of nodes. To maintain permanent communication connectivity, each sensor has to remain within a transmission range of at least one other node, regardless of the moving strategy. Moreover, mobility consumes a considerable amount of energy. The motion trajectories should ensure fast target point achievement with minimum energy consumption. Another problem to solve is how to guide mobile devices to explore entire workspaces, avoiding obstacles and reaching target positions. A comprehensive study of existing mobility models can be found in the literature [14,15,16,17,18]. Many of the algorithms for motion trajectory calculation have been introduced in mobile robotics [19,20] and next adapted to wireless mobile ad hoc networks.

### 2.2. Overview of Movement-Assisted Deployment Strategies

In general, various movement-assisted strategies have been investigated for sensor placement. They can be classified with respect to various criteria, such as the expected network configuration, operation mode and computing organization, the hardware’s capabilities, nodes’ properties, such as the ability for autonomous operation, etc. Surveys of existing solutions can be found in the literature [2,3,21,22].

With regard to the hardware capabilities of devices that form a network and fitting all or selected sensors with a mobility platform, we can distinguish two deployment schemes:

- sensors conveyed by single or several mobile platforms,
- mobile sensors.

In the first one, a single or several mobile platforms (robots or unmanned vehicles) carry sensors and move around a working scene. While traveling, they deploy sensors at desired positions; usually the vertices of a geographic grid. In the second strategy, it is assumed that each sensor has locomotion. Hence, a network comprises multiple autonomous mobile devices (mobile sensors) that can place themselves by changing their geographic location usually in a collaborative way. The advantage of this solution is the high flexibility: a network topology can easily adapt to a changing environment. Sensor self-deployment can take place after initial sensor dropping or a team of mobile sensors can migrate from the base to the target location.

In general, sensor migration can be accomplished in a direct or shifted manner. With regard to the manner of sensor migration, we can distinguish:

- one-stage deployment,
- multi-stage deployment.

In one-stage deployment (direct migration), the whole motion trajectory is built, and the sensor moves toward the target position. In multi-stage deployment (shifted migration), a group of sensors changes position simultaneously. A multi-hop motion pattern is calculated, and each sensor shifts its position by one hop toward the target location.

With regard to the calculation scheme and deployment process organization, we can distinguish:

- pre-defined deployment,
- self-organizing deployment,
- hybrid deployment.

**Figure 1:**
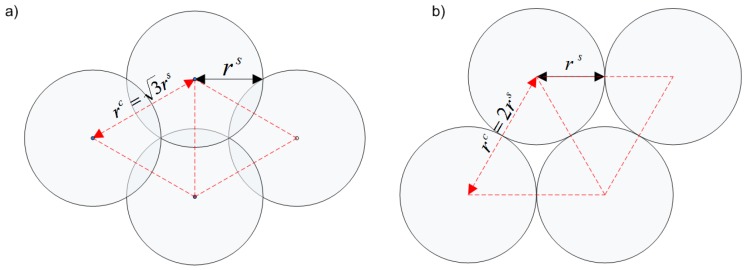
Regular grid topologies maintaining full coverage and connectivity ($r^{t}\geq r^{c}$): (**a**) square with overlapping; (**b**) triangular.

In pre-defined deployment, all spatial positions of sensors are a priori (off-line) determined by the central dispatcher and transmitted to the mobile sensors. Next, all sensors are forced to move, avoiding obstacles, in the advisable direction with adequate speed to reach the target positions. Finally, a pre-defined network topology is formed. In the simplest case, regular grid topologies are created. Various forms of such topologies have been considered and compared in the literature. The most popular one are presented in Figure 1. A comprehensive analysis of the widely-used triangular grid deployment is presented in [1].

Another approach is to calculate the optimal spatial positions of sensors taking into account the shape and characteristics of a sensing scene and phenomenon [10,23]. The covering optimization problem is formulated and solved by optimization solvers.

Self-organizing deployment is usually one possible option when the environmental dynamics precludes off-line pre-configuration, and nodes have to self-configure to establish an optimal network topology that maintains the sensing and communication coverage under energy constraints [3,24]. Hence, sensors are not assigned to fixed spatial positions, but are capable of tracking a changing environment. In this approach, all sensors that possess a certain amount of decision making autonomy move, avoiding obstacles in a workspace, while forming a multi-hop network that enables the continuous communication with the base station. Moreover, they perform cooperative simultaneous localization and communicate over the network. This strategy has important advantages over a scheme with pre-configuration, including autonomous deployment and flexibility. However, the realization of these envisaged gains depends on the communication and coordination capabilities of the network system.

Finally, to combine the advantages of pre-defined and self-organizing deployment, hybrid schemes have been developed. In these strategies, the deployment of sensors is composed of two (or more) stages. In the first stage, sensors are forced to move to advisable targets: nodes of the network determined by the central dispatcher. In the second stage, sensors are switched to the self-organizing deployment to adapt the network topology to the current snapshot of the sensing scene.

Considering the distribution of the decision process, we can distinguish:

- centralized deployment,
- distributed deployment,
- deployment with coordination.

In the centralized approach, the locations of all network nodes and motion trajectories are off-line calculated by the central dispatcher and transferred to sensing devices. In such an approach, the base station has to possess global knowledge about the whole working space and sensing phenomenon. It cannot be applied to monitoring processes with fast dynamics. In distributed deployment, all sensors are autonomous agents that collaborate to create a network. Each node estimates its own position based on the local data gathered from its neighbors and creates the motion trajectory. In the last approach, a two-level decision structure is implemented. The base station coordinates the sensor placement by influencing the individual sensors’ decisions.

## 3. Problem Statement

The aim of our research is to construct of a multi-hop wireless sensor network that enables sensing coverage of a bounded region of interest ROI⊂W and that intermittently transmits measurements to the base station. It is assumed that the network can operate in a disaster area, harsh fields, etc., and the sensing space ROI can move in *W* and change its shape. A WSN sensing system has to evolve and dynamically adapt its topology with respect to changes in the workspace. Hence, sensors cannot be deployed manually, and the movement-assisted deployment described in Section 2.1 is the only viable option.

### 3.1. Application Scenarios Definition

Consider a network WSN comprised by a set $S_{D}$ of *N* mobile devices $D_{i}$ (network nodes), [25,26]

(1) $$WSN=\left(S_{D},S_{L}\right),S_{D}\neq \emptyset ,\;\;S_{L}\neq \emptyset ,$$

where:

(2) $$\begin{array}{cc} S_{D} & =\{D_{i}:i=1,\ldots ,N\}, \end{array}$$

(3) $$\begin{array}{cc} S_{L} & =\{\left(D_{i},D_{j}\right):D_{i}\in S_{D},D_{j}\in S_{D},\;\;i,j=1,\ldots ,N,i\neq j\}. \end{array}$$

$S_{L}$ denotes the set of active direct connections between each pair of devices $D_{i}$ and $D_{j}$, i≠j. Each $D_{i}$ is equipped with the radio transceiver and the detector (or a set of detectors) and has locomotion. Hence, it is a solid body with any shape, which can move and rotate in a spatial domain $W\subset {ℜ}^{3}$. Its position is described by a reference point $c_{i}=\left[x_{i},y_{i},z_{i}\right]$, which is the location of its antenna, and its orientation is defined by the following Equation [19]:

(4) $$Q_{i}=q_{0_{i}}+\sum_{j=1}^{3}q_{j_{i}}k_{j},$$

where $Q_{i}$ denotes a quaternion $k_{1}$, $k_{2}$ and $k_{3}$ the fundamental quaternion units. Each $D_{i}$ can move with the speed $v_{i}\in \left[v_{i_{min}},v_{i_{max}}\right]$.

We define a set $S_{O}$ of obstacles in the workspace *W* that have to be avoided by mobile sensors. All obstacles are solid bodies with any shape, as well. Two types of obstacles are distinguished, i.e., $S_{O}=S_{M}\cup S_{S}$. A set $S_{M}$ consists of mobile obstacles $O_{m}$, m=1,…,M, and a set $S_{S}$ consists of static obstacles $O_{s}$, s=1,…,S.

Three application scenarios described in Section 2.2 are considered.

1. Pre-defined deployment: each network node is forced to move in the advisable direction to reach its target location $g_{i}=\left[x_{i}^{g},y_{i}^{g},z_{i}^{g}\right]$, $g_{i}\in ROI$. The target location can change over time.
2. Self-organizing deployment: all devices can move and organize themselves in an as large as possible network to cover the ROI taking into account their detectors and radio ranges.
3. Hybrid deployment: the combination of pre-defined and self-organizing strategies.

In the aforementioned scenarios, a satisfactory connectivity among all working sets of sensors and the base station should be provided. While forming a network for permanent monitoring and transmit measurements to the base station, the reference distances ${\hat{d}}_{ij}$ between $D_{i}$ and other nodes ($D_{j}$, j=1,…,N, j≠i) and the reference distance ${\hat{d}}_{ig}$ to the target point $g_{i}$ should encourage the creation of a well-connected network with good coverage. The performance measures for a good coverage and connectivity network are defined in the following subsection.

### 3.2. Performance Metrics

Multiple metrics can be used to measure the performance of a movement-assisted sensor placement. It is not enough to observe only coverage. Referring to the literature [1,6,23,27,28,29] and considering the results of our research, we have provided the following performance measures: connectivity, compact coverage, quality of transmission and cost. These are mainly caused by economic or technical constraints, such as hardware cost, low battery power and limited computation capabilities.

#### 3.2.1. Connectivity

The fundamental characteristic of a wireless sensor network is connectivity. Direct or indirect connections between all sensors and the base station are expected. In general, the measurements of all isolated nodes are useless. The WSN that maintains the permanent connection with the base station is comprised of a set $S_{D}^{C}$ of devices that form a topology satisfying the following condition:

(5) $$\begin{array}{ccc} S_{D}^{C} & = & \{D_{i}:\forall_{D_{i}\in S_{D}}\exists_{P_{i}^{p}=\left\{D_{1}^{p},\ldots ,D_{K}^{p}\right\}}d_{l,l+1}^{p}\leq r_{l}^{t},\;\;l=1,\ldots ,K-1\}, \end{array}$$

where *p* denotes an identifier of a transmission path from the device $D_{i}$ to the base station BS formed by a set $P_{i}^{p}$ of *K* ordered nodes, such that $D_{1}^{p}=D_{i}$, $D_{l}^{p}\in S_{D}^{C}$, $D_{l}^{p}\neq D_{i}$ and $D_{K}^{p}=BS$. $d_{l,l+1}$ and $r_{l}^{t}$ denote the distance between the node *l* and its successor in the path *p* and the transmission range of $D_{l}^{p}$, respectively.

Sensors without direct or indirect connection with the base station form a set of isolated nodes, $S_{D}^{I}\subset S_{D}$, $S_{D}^{C}\cup S_{D}^{I}=S_{D}$. Hence, the number of isolated nodes $C_{iso}$ in WSN is equal to $|S_{D}^{I}|$. It is obvious that the lesser the number of isolated nodes, the better the connectivity; therefore, $C_{iso}$ should be minimized.

#### 3.2.2. Compact Coverage

Compact coverage is the most important requirement of the sensors’ deployment. In most applications, it is expected to cover as large a part of a given region of interest as possible. Therefore, the percent of ROI covered by a WSN can be adopted as the performance metric. It is calculated as follows:

(6) $$C_{cov}=\frac{{⋃}_{D_{i}\in S_{D}^{C}}cov_{i}^{s}\cap ROI}{ROI}\cdot 100\%,$$

where ROI denotes a region of interest, $D_{i}$ the *i*-th sensing device (network node), $S_{D}$ a set of whole devices, $S_{D}^{C}$ a subset of $S_{D}$ defined in Equation (5) and $cov_{i}^{s}$ the sensing coverage of the *i*-th device.

The coverage is related to the number of sensing devices and their hardware equipment. Sometimes, particularly in large ROI and poorly-equipped devices, problems with ensuring coverage of good compactness may occur. In such a situation, a question is how much of the ROI can be sensed. In the case of poor results, the only option is to increase a number of nodes in a network.

#### 3.2.3. Quality of Transmission

In the case of many sensor networks based on contention-based MAC protocols (e.g., CSMA (Carrier Sense Multiple Access)), the quality of transmission is often decreased by an interference. It is obvious that the smaller the number of connections with neighboring nodes, i.e., the smaller node degree, the less interference is observed (see [29]). On the other hand, an insufficient number of connections with neighboring nodes endangers the network reliability, by limiting the number of alternative paths to the sink.

In order to improve the quality of transmission, either scheduled-based MAC protocols, like time-synchronized channel hopping (TSCH) [30,31], or networks with an appropriate node degree should be considered. Therefore, in our paper we propose to keep the average network node degree $C_{dim}$,

(7) $$C_{dim}=\frac{2|L|}{|S_{D}^{C}|}=\frac{1}{|S_{D}^{C}|}\sum_{D_{i}\in S_{D}^{C}}dim\left(D_{i}\right)$$

on the low level, not-exceeding 10 (the best results of the experiments were collected for $C_{dim}∼4-6)$. In the above equation *L* denotes a total number of active links in a whole network WSN and $dim(D_{i})$ the degree of the node *i*.

#### 3.2.4. Cost of Deployment

The obligation of providing high coverage and connectivity leads to the necessity in dealing with the network construction costs’ trade-off. We restricted our consideration only to the time of deployment and energy consumption, which are commonly listed costs of deployment. Moreover, we did not take into account costs concerned with the further activity of the created system, i.e., monitoring, tracking, etc.

Robots’ motion consumes the most energy and time. The energy used for wireless transmission is significantly smaller and can be ignored. Therefore, the path length of all mobile platforms $C_{len}$ from the starting points to the target points should be minimized.

(8) $$C_{len}=\sum_{t=1}^{C_{tim}}\sum_{D_{i}\in S_{D}}∥c_{i}^{\left(t\right)}-c_{i}^{\left(t-1\right)}∥,$$

where $C_{tim}$ denotes the total time of a given WSN deployment, *t* ($t=1,\ldots ,C_{tim}$) is a time step and $c_{i}$ is the reference point of the node $D_{i}$.

The criteria $C_{cov},C_{iso},C_{dim}$ can be used to assess the quality of a sensing system, i.e., sensing coverage, its connectivity and efficiency. $C_{len}$ and $C_{tim}$ show the energy and computational costs of the deployment process.

## 4. Collaborative Mobile Sensing Network Design

As was stated in Section 3, the aim is to create an optimal architecture of a mobile sensing network for indoor or outdoor ROI permanent monitoring. In systems that exploit mobile sensors, the spatial positions of all sensors in a workspace have to be constantly updated. Therefore, the important component of the deployment system is the mobility model for computing paths on which the platforms carrying sensors can move to a desired destination. We have developed such a model. Its preliminary version is described in [26]. In this section, we present and discuss the application of our mobility model to determine the collision-free motion trajectories for mobile sensors forming sensing systems for outdoor and indoor monitoring.

### 4.1. Algorithm for Optimal Displacement of Sensing Device Calculation

#### 4.1.1. Model of a Sensing Device

Consider a WSN as defined in Section 3 that operates in a workspace *W*. The network is expected to cover the region of interest ROI⊂W whose boundary is known and to provide consistent, quality sensing service. All measurements have to be continuously transmitted to the base station using multi-hop communication.

**Figure 2:**
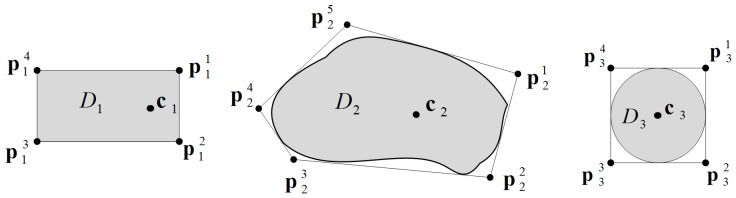
Models of regular- and irregular-shaped network nodes.

In order to simplify the description of WSN, we model each network node $D_{i}$ (i=1,…,N), obstacles $O_{m}$ (m=1,…,M) and $O_{s}$ (s=1,…,S) by a polyhedron, in which a given object is fully enclosed. $P_{i}$ denotes a set of $L_{i}$ vertices of the *i*-th polyhedron, $P_{i}=\left\{p_{i}^{1},\ldots ,p_{i}^{L_{i}}\right\}$. Models of regular and irregular network nodes in two-dimensional space are presented in Figure 2.

In general, radio and sensing coverage areas of each wireless sensing device are highly irregular, because of interference, obstacles, etc. However, including all of these aspects in communication and sensing models makes them extremely complex. Therefore, we model the radio coverage of $D_{i}$ by a disc $cov_{i}^{t}$ of a radius $r_{i}^{t}$ and the sensing coverage $cov_{i}^{s}$ by a disc of a radius $r_{i}^{s}$, both centered at the reference point $c_{i}$.

#### 4.1.2. Virtual Force Mobility Model

We have developed a novel virtual force model for sensor displacement calculation in a collaborative and coherent WSN. The virtual force technique was originally invented and used in mobile robotics [19]. It is based on the concept of an artificial potential field and an artificial potential function. The artificial potential field can be viewed as a landscape where a mobile device moves from a high-value state to a low-value state. The value of the artificial potential function *V* depends on a Euclidean distance *d* between the considered devices or objects. It is viewed as energy and is computed as a sum of repulsive and attractive potentials. The idea is that the target point attracts the mobile device, while the obstacle repels it. The virtual force is expressed by the gradient of *V*.

Consider WSN formed by *N* sensing devices $D_{i}$, i=1,…,N, acting in the workspace *W*. In general, the total potential between $D_{i}$ and all other objects in *W*, i.e., other nodes and all obstacles, can be expressed as follows:

(9) $$\begin{array}{c} V_{i}\left(d_{i}\right)=\sum_{j=1,j\neq i}^{N}V_{ij}\left(d_{ij}\right)+\sum_{s=1}^{S}V_{is}\left(d_{is}\right)+\sum_{m=1}^{M}V_{im}\left(d_{im}\right)+V_{ig}\left(d_{ig}\right), \end{array}$$

(10) $$\begin{array}{c} d_{ij}=\left|\right|c_{i}-c_{j}||,\;d_{im}=\left|\right|c_{i}-c_{m}||,\;d_{is}=\left|\right|c_{i}-c_{s}||,\;d_{ig}=\left|\right|c_{i}-g_{i}\left|\right|, \end{array}$$

where $V_{ij}$, $V_{is}$, $V_{im}$ and $V_{ig}$ denote the potential functions between the sensor $D_{i}$ and, respectively, the *j*-th sensor, the *s*-th static obstacle, the *m*-th mobile obstacle and the target point $g_{i}=\left[x_{i}^{g},y_{i}^{g},z_{i}^{g}\right]$, explicitly determined for the device $D_{i}$ (the goal of migration). $d_{ij}$, $d_{is}$, $d_{im}$ and $d_{ig}$ are Euclidean distances between the *i*-th sensor and, respectively, the *j*-th sensor, the *s*-th and the *m*-th obstacle, and the target points, $c_{i}$, $c_{j}$, $c_{s}$, $c_{m}$, are the reference points of $D_{i}$, $D_{j}$, $O_{s}$ and $O_{m}$ after a network transformation.

The main difficulty in mobility models utilizing the concept of the artificial potential field is to construct a potential function that can be used for on-line computing of a new location of a moving device and that does not introduce oscillations and many local minima not corresponding to the target of movement. Much research derives inspiration from classical and quantum mechanics. We developed a simple artificial potential function $V_{i}$ drawing on the Lennard–Jones potential commonly used to model the interactions between a pair of neutral atoms or molecules in liquid crystals [32]. The preliminary version was provided in [26]. The extended one, presented in this paper, was used to model the interactions between a given *i*-th node and all other objects in a workspace *W*, i.e., other network nodes and all obstacles.

(11) $$V_{i}\left(d_{i}\right)=\left[\sum_{j=1,j\neq i}^{N}\epsilon_{ij}{\left(\frac{{\hat{d}}_{ij}}{d_{ij}}-1\right)}^{2}+\sum_{s=1}^{S}\epsilon_{ij}{\left(\frac{{\hat{d}}_{is}}{d_{is}}-1\right)}^{2}+\sum_{m=1}^{M}\epsilon_{im}{\left(\frac{{\hat{d}}_{im}}{d_{im}}-1\right)}^{2}+\epsilon_{ig}{\left(\frac{{\hat{d}}_{ig}}{d_{ig}}-1\right)}^{2}\right].$$

**Figure 3:**
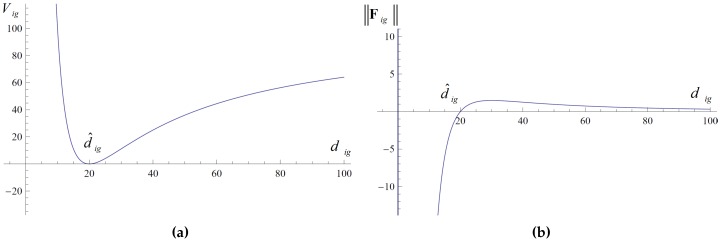
(**a**) The artificial potential function $V_{ig}$ between the sensor $D_{i}$ and its target point $g_{i}$; (**b**) the gradient of $V_{ig}$ (${\hat{d}}_{ig}=20$, $\epsilon_{ig}=1$).

In the above equation, $\epsilon_{ij}\geq 0$, $\epsilon_{is}\geq 0$, $\epsilon_{im}\geq 0$ and $\epsilon_{ig}\geq 0$ denote weighting factors determining the importance of, respectively, the device *j*, the obstacles *s*, *m* and the target *g*. $d_{ij}$, $d_{is}$, $d_{im}$ and $d_{ig}$ are distances calculated based on current measurements. ${\hat{d}}_{ij}$, ${\hat{d}}_{is}$, ${\hat{d}}_{im}$ and ${\hat{d}}_{ig}$ are the reference distances between $c_{i}$ and, respectively, $c_{j}$, $c_{s}$, $c_{m}$ and $g_{i}$. ${\hat{d}}_{ig}$, ${\hat{d}}_{is}$ and ${\hat{d}}_{im}$ are determined by a user, while ${\hat{d}}_{ij}$ is constantly updated due to current measurements. A sample $V_{ig}$ and its gradient (force) $F_{ig}=\nabla V_{ig}$ are depicted in Figure 3a,b. In general, all functions $V_{ij}$, $V_{is}$ and $V_{im}$, the other components of $V_{i}$ (Equation (9)), are similar in shape.

In the reference positions of all $D_{i}$ (i=1,…,N), i.e., $d_{ig}={\hat{d}}_{ig}$, $d_{ij}={\hat{d}}_{ij}$ (j=1,…,N), $d_{is}={\hat{d}}_{is}$ (s=1,…,S) and $d_{im}={\hat{d}}_{im}$ (m=1,…,M), we have an optimal network topology for a given application scenario (an unstable equilibrium ($V_{i}\approx 0$, i=1,…,N)).

However, it is obvious that in each time step, collisions of $D_{i}$ with only a few obstacles may occur. Therefore, the definition of the potential function (Equation (11)) can be simplified. Let us assume that in a given time $t_{k}$, only $H_{i}$ nearby obstacles (static and mobile) are located on the path to the target point $g_{i}$. Moreover, $b_{i}^{h}$ is the point from the edge of the *h*-th obstacle ($h=1,\ldots ,H_{i}$) with the shortest distance to $c_{i}$. The potential function (Equation (11)) can be substituted by the following one:

(12) $$V_{i}\left(d_{i}\right)=\left[\sum_{j=1,j\neq i}^{N}\epsilon_{ij}{\left(\frac{{\hat{d}}_{ij}}{d_{ij}}-1\right)}^{2}+\epsilon_{ig}{\left(\frac{{\hat{d}}_{ig}}{d_{ig}}-1\right)}^{2}+\sum_{h=1}^{H_{i}}\epsilon_{ih}{\left(\frac{v_{i_{max}}\cdot \Delta t}{d_{ih}}-1\right)}^{2}\right],\;i=1,\ldots ,N.$$

In this definition of the potential function, the reference distance to the detected obstacle is equal to $v_{i_{max}}\cdot \Delta t$. $d_{ih}=∥c_{i}-b_{i}^{h}∥$ denotes a distance to the *h*-th obstacle estimated in the time step $t_{k}$.

Finally, we can calculate the expected position of the reference point $c_{i}$ of the device $D_{i}$ and all points, vertices of its envelope, $p_{i}\in P_{i}$, solving the optimization problem:

(13) $$\min_{c_{i},p_{i}^{1},\ldots ,p_{i}^{L_{i}}}\left[\sum_{j=1,j\neq i}^{N}\epsilon_{ij}{\left(\frac{{\hat{d}}_{ij}}{d_{ij}}-1\right)}^{2}+\epsilon_{ig}{\left(\frac{{\hat{d}}_{ig}}{d_{ig}}-1\right)}^{2}+\sum_{h=1}^{H_{i}}\epsilon_{ih}{\left(\frac{v_{i_{max}}\cdot \Delta t}{d_{ih}}-1\right)}^{2}\right],\;i=1,\ldots ,N.$$

under the constraints:

(14) $$\forall_{\begin{array}{c} p_{i}^{a}\in \left\{p_{i}^{1},\ldots ,p_{i}^{L_{i}}\right\} \end{array}}\;∥c_{i}-p_{i}^{a}∥=const,$$

(15) $$v_{i}\in \left[v_{i_{min}},v_{i_{max}}\right].$$

The constraints specified in Equation (14) assure that the shape of each device will be preserved after displacement (Figure 2). Due to assumption that each network node is a solid body, the distances between different points from the set $P_{i}$ and the point $c_{i}$ remain constant. The constraint (15) determines the speed range.

The optimization problem Equations (13)–(15) has to be solved repetitively, every time step $t_{k}$, ($t_{k+1}-t_{k}=\Delta t$) for all network nodes $D_{i}$, i=1,…,N. After computing new optimal locations, all nodes are moved to these locations avoiding all nearby obstacles.

#### 4.1.3. Algorithm for Collision-Free Traversing Pattern Calculation

A virtual force algorithm was developed to calculate a collision-free traversing pattern from one WSN configuration to another. In general, the algorithm executed autonomously by each $D_{i}$ is composed of three main steps executed repetitively:

Step 1: Estimation of the reference internode distances ${\hat{d}}_{ij}$ (i=1,…,N, j=1,…,N, i≠j) based on the current sensing and transmission ranges of all $D_{i}$.
Step 2: Calculation of the new positions of $D_{i}$ solving the optimization problem Equations (13)–(15) with the performance measures equal to a value of an artificial potential function.
Step 3: Relocation of $D_{i}$ to the determined position avoiding all nearby obstacles.

To estimate the reference distance ${\hat{d}}_{ij}$ between two neighboring nodes that implies both coverage and connectivity, the current transmission range $r_{i}^{t}$ and sensing range $r_{i}^{s}$ have to be taken into consideration. The sensing range is usually specified for a given detector; the transmission range $r_{i}^{t}$ is usually specified for a given transceiver; but in many application scenarios, they should be adjusted to communication the conditions and working scene. Current internode distances $d_{ij}$ and reference distances ${\hat{d}}_{ij}$ can be calculated based on known geographical locations of nodes and current measurements. In general, the measurements can be given by various components of each device, e.g., radio transceivers, cameras, detectors, etc. In the case studies, results of which are presented in this paper, a commonly-used radio signal propagation model and signal strength measurements based on RSSI (received signal strength indicator) were applied to estimate the current and reference internode distances. Namely, we used a long-distance path loss model for the estimation of signal degradation with a distance $d_{ij}$ [33]:

(16) $$Pow_{i}^{t}\left(d_{ij}\right)\left[dBm\right]=Pow_{j}^{r}\left[dBm\right]-PL\left(d_{ij}\right)\left[dB\right],$$

where $Pow_{i}^{t}$ denotes the power used by the *i*-th node to transmit the signal, $Pow_{j}^{r}$ the power of the signal received by the receiver (*j*-th node) and $PL(d_{ij})$ (path loss) the average degradation of a signal with a distance $d_{ij}$, defined as follows:

(17) $$PL\left(d_{ij}\right)\left[dB\right]=PL\left(d_{0}\right)\left[dB\right]+10qlog\left(\frac{d_{ij}}{d_{0}}\right)+X_{\sigma },$$

where *q* is an attenuation constant, which indicates the rate at which the path loss increases with a distance, $d_{0}$ denotes a reference distance ($d_{0}$ = 1 m for IEEE 802.15.4 [30]) and $X_{\sigma }$ a zero-mean Gaussian distributed random variable with standard deviation *σ* (all in dB).

In the practical scenarios, the internode distances and reference distances can be estimated using the Equations (16) and (17) model. The least square method and RSSI measurements can be applied to calculate all parameters of this model. The well-known Q-function in statistics described in [33] can be used to determine the probability that the received signal level will exceed the sensitivity of the transceiver. The detailed description of internode distances’ estimation can be found in [12,26].

After reference internode distance estimation, each $D_{i}$ computes its expected location in a workspace taking into account these distances, the target destination (if it is recommended) and possible collisions with obstacles in *W*. In the time steps $t_{1},t_{2},\ldots ,t_{K}$, each *i*-th device calculates its new position in *W* solving the optimization problem Equations (13)–(15). Next, it moves to the designated location. The tangent bug algorithm described in [19] can be applied to avoid obstacles. The calculations are repeated every time interval Δt due to changes in communication conditions and the workspace. The detailed description of all steps of the algorithm is provided in [26].

### 4.2. Deployment Strategies and Computing Schemes

We have developed algorithms for pre-defined and self-organizing deployment, both utilizing our mobility model for platforms carrying sensors traversing pattern calculation. In general, in the pre-defined deployment, the optimal positions of $D_{i}$, i=1,…,N in *W* are explicitly off-line calculated; while, in the self-organizing deployment, the target positions are calculated on-line and are dynamically updated.

In our deployment strategies, we have employed two computing and decision schemes: distributed and two-level with a supervisor. In the first one, all calculations are performed by network devices based on locally-available information. In the second one, a central dispatcher (base station) influences the decisions of these devices. The central dispatcher computes its decisions based on data about a workspace and the current states of all sensing devices.

#### 4.2.1. Pre-Defined Deployment

A coherent sensing system with a triangular topology has been investigated. Taking into account the sensing and transmission ranges, the maximum distance $d_{ij}^{max}$ between pairs of neighbors (*i*-th and *j*-th) in a triangular topology that implies both sensing coverage and connectivity is as follows:

(18) $$d_{ij}^{max}=\left\{\begin{array}{cc} \sqrt{3}\min\left(r_{i}^{s},r_{j}^{s}\right), & \min\left(r_{i}^{t},r_{j}^{t}\right)\geq \sqrt{3}\min\left(r_{i}^{s},r_{j}^{s}\right),\\ \min(r_{i}^{t},r_{j}^{t}), & \mathrm{otherwise}. \end{array}\right.$$

The two-level computing scheme can be employed to calculate the target positions of mobile robots carrying sensors and traversing patterns to these points. First, the base station explicitly calculates from Equation (18) the reference internode distances, ${\hat{d}}_{ij}=d_{ij}^{max}$, i,j=1,…,N, i≠j. Next, the desired locations of the reference points of all network nodes ${\hat{c}}_{i}$, i=1,…,N are computed. The target positions and reference distances are distributed among robots carrying sensors. Finally, all robots calculate the optimal traversing patterns to carry sensors to the desired locations. Every time interval Δt, each robot computes its new position in the advisable direction solving one of the following optimization problems:

- Variant A: unknown map of *W* (unknown locations of obstacles).
  (19) $$\min_{c_{i},p_{i}^{1},\ldots ,p_{i}^{L_{i}}}\left[{\left(\frac{{\hat{d}}_{ig}}{d_{ig}}-1\right)}^{2}\right],$$
- Variant B: known map of *W* (full knowledge about all obstacles is provided).
  (20) $$\min_{c_{i},p_{i}^{1},\ldots ,p_{i}^{L_{i}}}\left[{\left(\frac{{\hat{d}}_{ig}}{d_{ig}}-1\right)}^{2}+\sum_{h=1}^{H_{i}}\epsilon_{ih}{\left(\frac{v_{i_{max}}\cdot \Delta t}{d_{ih}}-1\right)}^{2}\right],$$

both under the constraints (14) and (15). Note that in the above objective functions, we have taken the weighting factor $\epsilon_{ig}=1$, i,g=1,…,N. Manipulating with the values of these factors, we can generate topologies with other shapes than the triangular one. Next, all robots are forced to move to new positions avoiding obstacles in *W*. The operations are repeated up to the robots reaching the target positions. It is obvious that in the case of a working scene with numerous obstacles, the final topology can be irregular in subareas.

#### 4.2.2. Self-Organizing Deployment

In self-organizing deployment, all robots carrying sensors move, avoiding obstacles in a workspace, while forming a coherent multi-hop network that enables the continuous communication with the base station, and cover the region of interest. In this computing scheme, robots are autonomous devices, and no central unit or supervisor influences their decisions. All calculations are fully distributed and on-line performed by network nodes. First, the estimates of reference distances ${\hat{d}}_{ij}$, i,j=1,…,N, i≠j are calculated from Equation (17) based on current geographical locations and the measurements of radio signal strength gathered from node’s neighbors, as well as with regard to the current communication conditions. To calculate these estimates, nodes have to perform cooperative simultaneous localization and communicate over the network.

Next, every time step $t_{k}$, the *i*-th node solves the following optimization problem and determines its desired location in the time step $t_{k+1}$. Similarly to pre-defined deployment, two variants are considered:

- Variant A:
  (21) $$\min_{c_{i},p_{i}^{1},\ldots ,p_{i}^{L_{i}}}\left[\sum_{j=1,j\neq i}^{N}\epsilon_{ij}{\left(\frac{{\hat{d}}_{ij}}{d_{ij}}-1\right)}^{2}\right],$$
- Variant B:
  (22) $$\min_{c_{i},p_{i}^{1},\ldots ,p_{i}^{L_{i}}}\left[\sum_{j=1,j\neq i}^{N}\epsilon_{ij}{\left(\frac{{\hat{d}}_{ij}}{d_{ij}}-1\right)}^{2}+\sum_{h=1}^{H_{i}}\epsilon_{ih}{\left(\frac{v_{i_{max}}\cdot \Delta t}{d_{ih}}-1\right)}^{2}\right],$$

under the constraints (14) and (15).

Next, each robot is forced to move to a new position, avoiding collisions. Similarly to the pre-defined deployment, the optimization problem is solved every Δt. However, in this deployment scheme, the reference distances have to be updated with respect to changes in the workspace.

The self-organization allows one to create topologies with various characteristics, dedicated to given application scenarios. Moreover, note that the final topology depends on the values of weighting factors $\epsilon_{ij}$ in the objective functions (21) and (22). For $\epsilon_{ij}=1$, i,j=1,…,N, the optimal topology should provide the maximum possible coverage of a given ROI. Manipulating with weighting factors, we can generate topologies with various densities of nodes in subregions.

This strategy has important advantages over a scheme with pre-configuration, including autonomous deployment and flexibility. However, the realization of these envisaged gains depends on the communication capabilities of the network system. Moreover, it can be inefficient in the case of workspace with narrow passages and multiple densely-deployed obstacles.

#### 4.2.3. Hybrid Deployment

This strategy combines the advantages of pre-defined and self-organizing deployment. Two variants of hybrid deployment have been developed and investigated. In both of them, the scheme for computing the optimal placement of sensing devices was divided into two stages.

Hybrid 1: concentration of nodes in a given subregion and switching into self-organizing deployment.
Hybrid 2: improvement of combining pre-defined deployment using the self-organizing capabilities of sensing devices.

In the first variant, all robots are forced to move to a neighborhood of one or several points in a workspace determined by the supervisor. Next, they are switched to the self-organizing deployment. Such an approach can be a good solution in the case of narrow passages in a workspace.

In the second variant, robots carrying sensors are forced to move to advisable targets: nodes of the grid determined by the base station. Next, they are switched to the self-organizing deployment. Thus, the regular topology adapts to the working scene. Such a deployment scheme can be especially useful in the case of multiple irregular obstacles in a workspace.

## 5. Case Study Results

### 5.1. Simulation Setup

A series of simulation experiments was conducted to validate and demonstrate the capabilities of all presented deployment strategies. In the last few decades, numerous software tools for ad hoc network simulation have been developed. The popular commercial and publicly-released simulators (e.g., ns-3 [34], TOSSIM [35], Cooja [36]) focus on the simulation of wire and wireless transmission. Tools for mobile robot motion simulation are provided by simulation environments for mobile robotics (e.g., V-Rep [37]). However, they offer a limited number of mobility models. Therefore, for the sake of results comparability, the formation of two-dimensional mobile wireless sensor networks for indoor and outdoor monitoring was implemented in the form of simulators developed using MobAsim [38], our tool for fast prototyping and simulation of ad hoc networks. The tasks for a team of mobile robots carrying sensors were to create high coverage and connectivity wireless sensor networks and minimize the cost of these networks’ construction. Results were compared with respect to the criteria defined in Section 3.2.

The collision-free motion patterns for all robots were calculated due to the mobility model described in Section 4.1. Two scenarios were considered, i.e., Variant A, the option without the a priori available map of ROI, and Variant B, the option with the known map of ROI. In Variant A, all obstacles were identified on-line by robots, while in Variant B, each robot used a map of terrain and obstacles for motion trajectory calculation.

### 5.2. Scenario 1: Indoor Environment Monitoring

**Figure 4:**
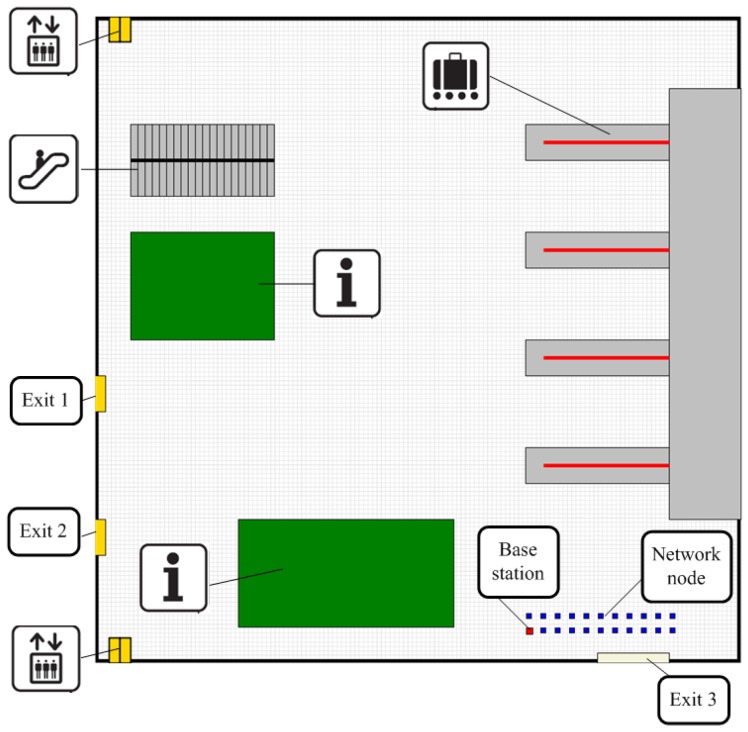
The arrival hall at the airport: sensing scene (ROI) and initial network topology.

The first case study was to design and develop wireless sensor networks for indoor environment monitoring. The aim was to cover the arrival hall (90 m × 90 m) at the airport by sensing devices and ensure permanent multi-hop communication between each device and the base station. Wireless networks with several configurations, composed of 22 mobile robots equipped with sensors and moving with a speed v∈ [0.2 m/s, 2 m/s], were created for sensing this hall. All robots were modeled by circles with the reference points $c_{i}$, i=1,…,22 at their centers. The initial positions of all robots were in the bottom right corner of the hall; see Figure 4. The robots communicated wirelessly sharing the same radio channel; the transmission range of all network devices was equal to $r^{t}=27.6$ m, while the sensing range was equal to $r^{s}=10$ m. Taking into account the size of the workspace and the limitations of sensing devices and radio transceivers the, maximum possible sensing coverage $C_{cov}$ of the obstacle-free ROI was equal to 85%.

**Figure 5:**
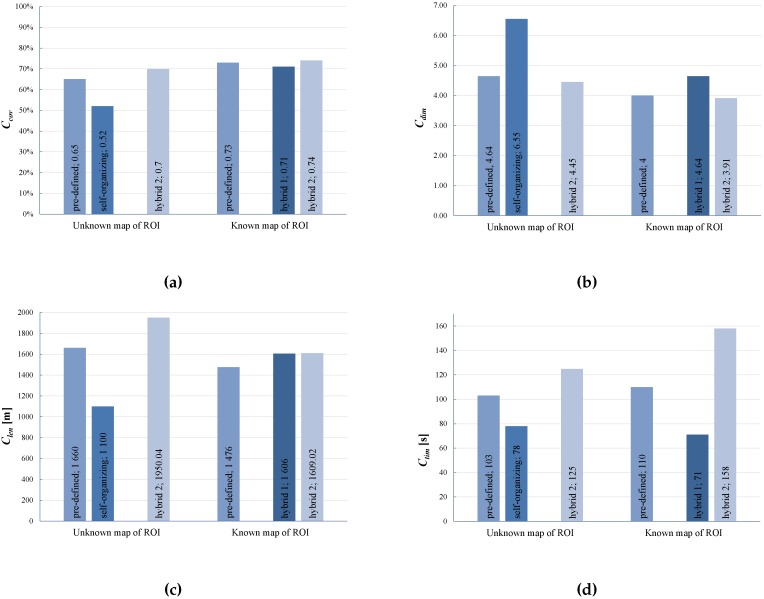
Comparison of the metrics values for pre-defined, self-organizing and hybrid deployment strategies applied in Scenario 1: (**a**) compact coverage; (**b**) average network node degree; (**c**) path length of mobile platforms; (**d**) time of deployment.

**Figure 6:**
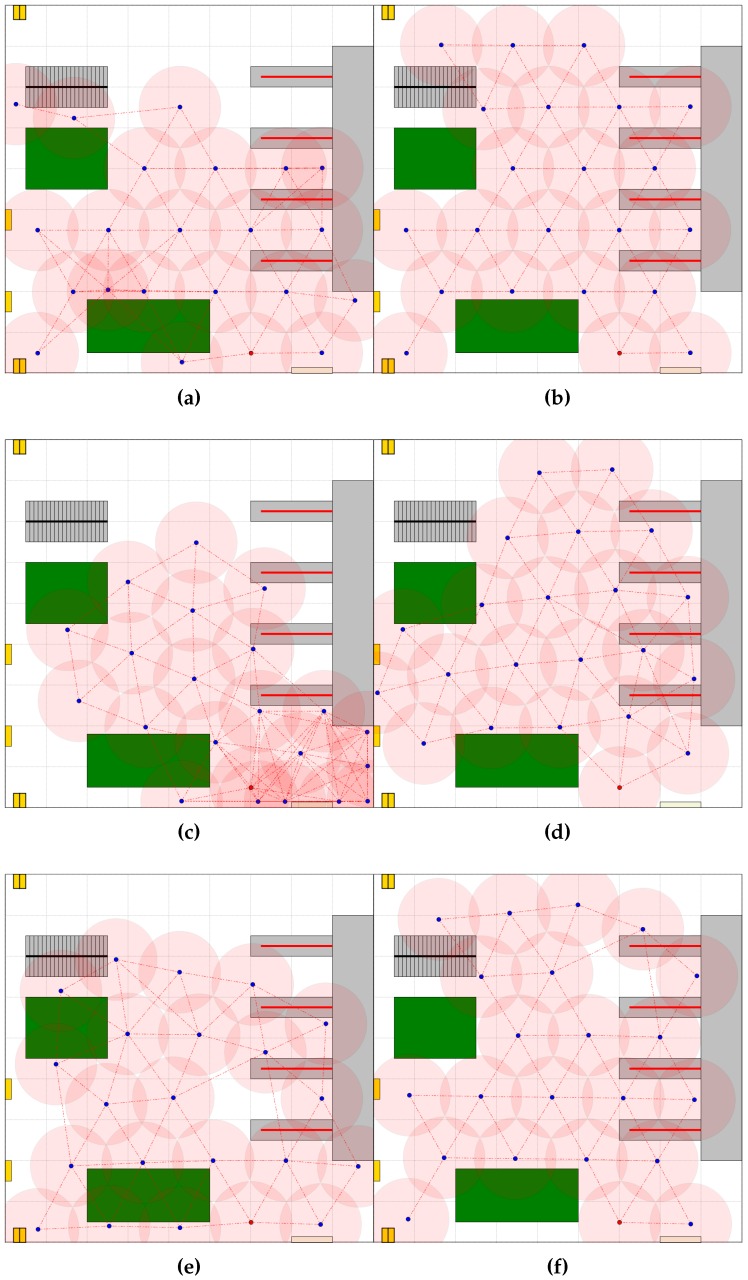
Created network topologies, various deployment strategies: (**a**,**b**) pre-defined (Variants A and B); (**c**) self-organizing (Variant A); (**d**) hybrid deployment (Hybrid 1, Variant B); (**e**,**f**) hybrid deployment (Hybrid 2, Variants A and B).

The values of the metrics defined in Section 3.2 and calculated for all developed sensing networks are collected in Table 1 and Figure 5. The results of the simulation of the 300 s of the network formation process exploiting pre-defined, self-organizing and hybrid deployment strategies with the known and the unknown map of ROI are presented in Figure 6a–f.

The first series of experiments was to create triangular grid topologies. In this scenario, the final exact positions of all sensors were calculated off-line by the central dispatcher in the base station and distributed to all robots. Next, the robots were forced to move to these positions.

In all experiments, high connectivity networks were obtained; Figure 6a,b. However, in the case of the variant with the unknown map of ROI, the obstacles forced some changes in the established regular topology and influenced both the coverage and cost of the network. The final coverage was equal to 65%; the total path length was equal to 1660 m; the time of the network deployment was equal to 103 s. Providing the map of ROI seriously improved the quality of the sensing network (Figure 6b). The coverage increased to 73%, and the total path length was reduced to 1475 m.

Next, the self-organizing deployment was tested. The robots carrying sensors were free to move, communicate and organize themselves. The goal was to form a high quality network. Figure 6c illustrates the network topologies obtained after 300 s of the network formation process in the case of the unknown map of ROI. Unfortunately, providing the map of ROI only slightly improved the quality of the sensing network. It can be seen that the performance metrics calculated for the self-organizing deployment are worse than the metrics obtained for the pre-defined deployment. The coverage decreased by 13% in the case of the unknown map. It was observed that deterioration in the final deployment was mainly due to the narrow passage in the workspace considered. Therefore, to improve the quality of the sensing network, the two-phase deployment (Hybrid 1) was used. The result of the application of this deployment strategy for a workspace with known obstacles is presented in Figure 6d. In the first phase, the intermediate target position was calculated by the central dispatcher and broadcast to all other devices. The robots were forced to move to reach the neighborhood of this position. Next, they freely organized themselves and formed the final network. The application of such a strategy allowed improving the final coverage to 71%. However, the final coverage was still worse than that obtained employing the pre-defined scheme for network topology calculation, but the time of deployment was seriously reduced.

**Figure 7:**
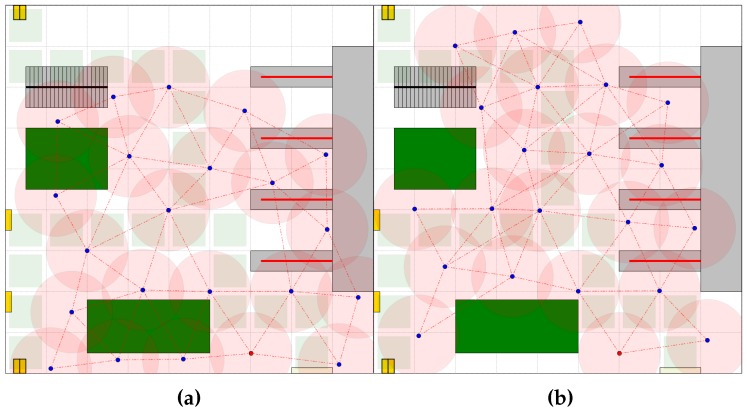
Created network topologies. Hybrid 2 deployment strategy: (**a**) Variant A; (**b**) Variant B.

Finally, the Hybrid 2 deployment strategy was evaluated through simulation. The sensing system was built in two phases. In the first phase the triangular grid topology network was created. The pre-defined deployment strategy was utilized. In the second phase, the self-organizing capabilities of the network nodes were applied to adapt the topology to ROI. The results of the simulation of 300 s of the network formation process are presented in Figure 6e,f. Adding self-organization in the final stage of the network formation process, we increased the coverage to 70% (Variant A) and 74% (Variant B). Obviously, the total path and time of deployment increased. In the last series of experiments, the results of which are depicted in Figure 7a,b, the network topologies were created under the assumption that passages for people and goods had to be free from sensors. The Hybrid 2 deployment strategy was used to create the monitoring network.

### 5.3. Scenario 2: Outdoor Environment Monitoring

**Figure 8:**
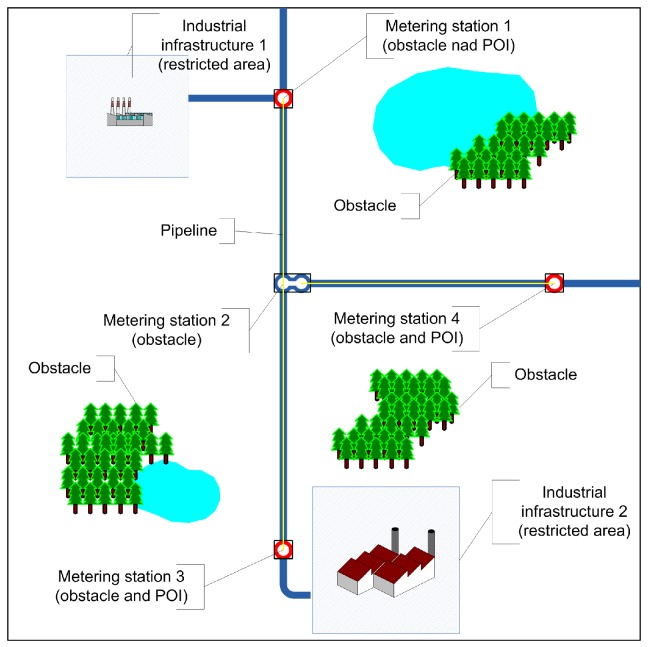
The workspace and the pipeline system under monitoring.

**Figure 9:**
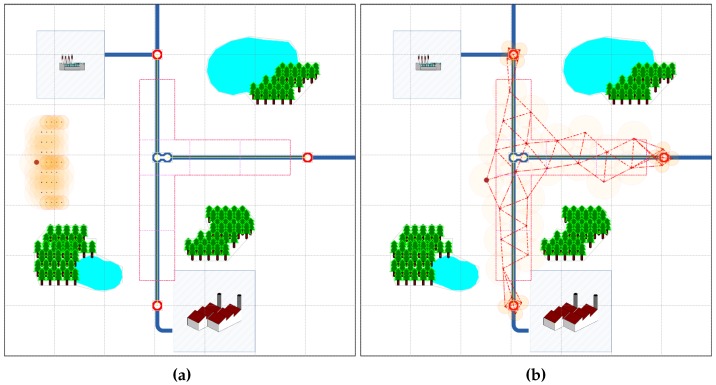
Topology of the sensing network: (**a**) initial topology; (**b**) final topology.

The aim of the second series of tests was to form a wireless sensor network for monitoring a gas pipeline system comprised of underground and ground infrastructure, i.e., pipes and four metering stations. The system was located in a square area (70 m × 70 m); Figure 8. The sensing area (ROI) was divided into six subregions; Figure 9a. A set $S_{D}$ of 20 mobile robots equipped with sensors and moving with the speed v∈ [0.2 m/s, 2 m/s] was used to monitor this gas pipeline system. Similarly to Scenario 1 all robots were modeled by circles with the reference points $c_{i}$, i=1,…,20 at their centers. The set $S_{D}$ was divided into six teams $S_{D}^{w}$, $S_{D}={⋃}_{w=1,\ldots ,6}S_{D}^{w}$; four consisting of three robots and two consisting of four robots; each team for the monitoring of one subregion. The Hybrid 2 deployment strategy was employed to create the sensing system.

The network formation process was evaluated through simulation. The transmission range of each transceiver was equal to $r^{t}=27.6$ m, and the sensing range was equal to $r^{s}=10$ m. First, the centers of all sections $g_{w}$, w=1,…,6 were determined by the central dispatcher based on the map of ROI, and the reference distances ${\hat{d}}_{iw}$, for all robots from each team $S_{D}^{w}$, were calculated according to Equation (18). Next, the target positions for all robots from all teams $S_{D}^{w}$, w=1,…,6, were computed by the central dispatcher solving the following optimization problem for ${\hat{d}}_{iw}=15$:

(23) $$\min_{c_{i},D_{i}\in S_{D}^{w}}\left[{\left(\frac{{\hat{d}}_{iw}}{d_{iw}}-1\right)}^{2}+\sum_{h=1}^{H_{i}}\epsilon_{ih}{\left(\frac{v_{i_{max}}\cdot \Delta t}{d_{ih}}-1\right)}^{2}\right],\;w=1,\ldots ,6,$$

under the constraints (14) and (15). In the above formulation, $d_{iw}=∥c_{i}-g_{w}∥$. The solution of the optimization (23), i.e., a vector of target points, was forwarded to the robot teams. Robots from each team were forced to move to reach the neighborhood of their target positions.

Next, all robots switched to the self-organizing deployment strategy. New target positions were repetitively calculated. The following optimization problem was solved autonomously by each robot from a team *w*:

(24) $$\min_{c_{i},D_{i}\in S_{D}^{w}}\left[\sum_{D_{j}\in S_{D}^{w},j\neq i}\epsilon_{ij}{\left(\frac{{\hat{d}}_{ij}}{d_{ij}}-1\right)}^{2}+\sum_{h=1}^{H_{i}}\epsilon_{ih}{\left(\frac{v_{i_{max}}\cdot \Delta t}{d_{ih}}-1\right)}^{2}\right],$$

under the constraints (14) and (15). The final coverage of the pipeline system under monitoring was equal to 98%, and the average network node degree was equal to 4.24. All metering stations were under monitoring. The total path length of all robots was equal to 12,635.78 m, and the time of the network deployment was equal to 301 s.

It is worth mentioning that the case study results presented in this paper are limited to the design of coherent and compact networks for monitoring. The proposed technique for the design of a collaborating mobile network employing our mobility model can be successfully applied to other scenarios. Numerous simulation experiments starting from simple to more complex wireless ad hoc networks topologies were conducted. Some of them are described and discussed in [26].

## 6. Conclusions

This paper has provided a short overview of wireless sensors’ deployment and coverage to monitor indoor and outdoor environment. We outlined the main properties and criteria that should be considered while creating the optimal network topologies for mobile sensing. Moreover, the paper has summarized the results of our research concerned with the application of optimization and simulation technologies to the on-line deployment of mobile autonomous wireless sensors for monitoring purposes. A family of algorithms utilizing pre-defined and self-organizing computing schemes for designing sensing network topologies maintaining permanent connectivity with the central dispatcher were developed and compared. They employ a novel mobility model for the optimal sensors’ displacement calculation.

The obtained results confirmed that pre-defined deployment is the best solution for the construction of a network for known, obstacle-free workspace monitoring. In the case of more realistic scenarios, when the aim is to monitor a scene with on-line-detected obstacles and moving objects, the hybrid strategy combining both pre-defined and self-organizing deployment is recommended. Including autonomous deployment and self-organizing capabilities of network nodes can improve the quality of the sensing topology, particularly in the case of an unknown map of the workspace considered. The main advantages of self-organizing and hybrid deployments are their flexibility and robustness. The sensing network may easily adapt to a given application scenario, especially in the case of a workspace with narrow passages, numerous unknown obstacles and dynamic changes in the sensing environment. Moreover, the network topology can be quickly modified as needed. Unfortunately, distributed self-organizing deployment involves high communication and computational costs and then a high energy consumption. In many practical applications, the limited resources of real-life networks comprised of simple devices can constrain us to use low complexity deployment strategies. Although numerous deployment methods and algorithms have been proposed and described in the literature, the development of a robust and scalable technique for the optimal placement of sensors in a working space with high sensing coverage, minimal hardware cost and computational burdens is still a challenging problem.

The presented strategies for mobile sensing network design have been already verified and evaluated through extensive simulations. Moreover, they will be validated based on the experiments carried out in the testbed networks. Currently, we are working on the prototype ad hoc network for supporting emergency situation awareness and rescue actions composed of mobile devices equipped with the radio transceiver nRF51822 manufactured by Nordic Semiconductors. The self-organizing and hybrid deployment strategies described and evaluated in this paper will be used to calculate the evolving positions of the devices. The next application considered is the implementation of a mobile monitoring system in the laboratory in cooperation with the Robotic Group at Warsaw University of Technology or in one of the experimental platforms (e.g., FIT IoT-LAB [39]). The system will be created from multiple robots carrying sensors and following motion trajectories calculated according to the algorithms described in this paper.

## Figures and Tables

**Table 1:** Performance metrics for various deployment strategies, known and unknown map of ROI.

Deployment Strategy	Unknown Map of *ROI* (Variant A)					Known Map of *ROI* (Variant B)				
	$C_{\mathrm{cov}}$	$C_{\mathrm{iso}}$	$C_{\dim}$	$C_{\mathrm{len}}\;\left(m\right)$	$C_{\mathrm{tim}}\;\left(s\right)$	$C_{\mathrm{cov}}$	$C_{\mathrm{iso}}$	$C_{\dim}$	$C_{\mathrm{len}}\;\left(m\right)$	$C_{\mathrm{tim}}\;\left(s\right)$
pre-defined	65	0	4.64	1659.55	103	73	0	4.00	1475.60	110
self-organizing	52	0	6.55	1099.57	78	-	-	-	-	-
Hybrid 1	-	-	-	-	-	71	0	4.64	1605.58	71
Hybrid 2	70	0	4.45	1950.04	125	74	0	3.91	1609.02	158
